# m-Calpain is required for preimplantation embryonic development in mice

**DOI:** 10.1186/1471-213X-6-3

**Published:** 2006-01-24

**Authors:** Previn Dutt, Dorothy E Croall, J Simon C Arthur, Teresa De Veyra, Karen Williams, John S Elce, Peter A Greer

**Affiliations:** 1 Division of Cancer Biology and Genetics, Queen's University Cancer Research Institute, Queen's University, Kingston, Ontario; K7L 3N6, Canada; 2 Department of Pathology and Molecular Medicine, Queen's University, Kingston, Ontario; K7L 3N6, Canada; 3 Department of Biochemistry, Queen's University, Kingston, Ontario; K7L 3N6, Canada; 4 Department of Biochemistry, Microbiology and Molecular Biology, University of Maine, Orono, Maine, 04469-5735 USA; 5 MRC Phosphorylation Unit, University of Dundee, DD1 5EH, UK

## Abstract

**Background:**

μ-calpain and m-calpain are ubiquitously expressed proteases implicated in cellular migration, cell cycle progression, degenerative processes and cell death. These heterodimeric enzymes are composed of distinct catalytic subunits, encoded by *Capn1 *(μ-calpain) or *Capn2 *(m-calpain), and a common regulatory subunit encoded by *Capn4*. Disruption of the mouse *Capn4 *gene abolished both μ-calpain and m-calpain activity, and resulted in embryonic lethality, thereby suggesting essential roles for one or both of these enzymes during mammalian embryogenesis. Disruption of the *Capn1 *gene produced viable, fertile mice implying that either m-calpain could compensate for the loss of μ-calpain, or that the loss of m-calpain was responsible for death of *Capn4*^-/- ^mice.

**Results:**

To distinguish between the alternatives described above, we deleted an essential coding region in the mouse *Capn*2 gene in embryonic stems cells and transmitted this mutant allele through the mouse germline. Breeding of heterozygous animals failed to produce homozygous mutant live offspring or implanted embryos. A nested PCR genotyping protocol was established, and homozygous preimplantation mutant embryos were detected at the morula but not at the blastocyts stage.

**Conclusion:**

We conclude that homozygous disruption of the *Capn2 *gene results in pre-implantation embryonic lethality between the morula and blastocyst stage. This establishes that μ-calpain and m-calpain have distinct functions, and that m-calpain is vital for development of the preimplantation murine embryo.

## Background

The two ubiquitous Ca^2+^-dependent, cysteine proteases known as μ-calpain (calpain-1) and m-calpain (capain-2), are the founding members of a gene family comprising 13 genes in mammals [1-3]. Both are heterodimeric enzymes consisting of distinct 80 kDa catalytic subunits, encoded by the *Capn1 *(μ-80 k) and *Capn2 *(m-80 k) genes, respectively, that associate with a common 28 kDa regulatory subunit encoded by the *Capn4 *gene. The μ-80 k and m-80 k subunits share 62% amino acid sequence identity, and are very similar in terms of structure, protein chemistry, and *in vitro *substrate specificity. Despite these similarities, the differential expression patterns of μ- and m-calpain in mammalian tissues suggest they have some isoform specific and distinct functions. The μ and m designations derive from the levels of Ca^2+ ^required *in vitro *for optimal activation; 10–50 μM Ca^2+ ^for μ-calpain and 0.3–0.35 mM Ca^2+ ^for m-calpain. It is generally assumed that μ- and m-calpain maintain their differential sensitivities to calcium *in vivo*, although this has not yet been strictly demonstrated. Furthermore, since the cytoplasmic free Ca^2+ ^concentration is typically less than 1 μM, it is also assumed that other *in vivo *factors must contribute to regulation of these enzymes [3].

Without apriori knowledge of the factors regulating calpain activity or their relevant substrates, elucidation of biological functions for calpains presents a challenge. Research on calpains has linked them with a wide variety of functions including muscle growth, development, degeneration (3), neuronal growth and neurodegeneration [4], cell cycle progression [5,6], signal cascades triggered by integrins and growth factors [7], membrane protrusion [8], remodeling of the cytoskeleton and cell migration [9-15], and regulation of cell death via both necrosis and apoptosis [16-22]. To date, the literature suggests a complex interplay between caspases and calpains [23,24] and impact of calpain on cell death pathway components [25]. The lack of highly specific cell-permeable inhibitors of calpains contributes to the challenge of investigating and defining calpain functions in these processes. Although over-expression of calpastatin, the endogenous protein inhibitor of μ- and m-calpain provides an important approach for these efforts, it will not distinguish isoform specific functions [24,26,27]. Some work has suggested isoform specific roles, such as a role for m-calpain in epidermal growth factor (EGF)-induced cell motility [28,29] and a role for μ-calpain in interferon-inducible protein 9-induced migration of keratinocytes [28]. A cell permeable calpain inhibitor (which likely inhibits other thiol-proteases as well) has been used to select cells lacking μ-calpain which display reduced proliferation rates [30]. Interestingly, m-calpain expression persisted in these cells, suggesting a possible requirement of m-calpain for cell survival [30].

Targeted gene deletion in mice provides a powerful approach to determining the physiological roles of μ- and m-calpain and the opportunity to approach their isoform specific functions. Initial studies targeted *Capn4 *based on the prediction that loss of this calpain subunit would abolish activity of both μ- and m-calpain. *Capn4*^-/- ^murine embryos died between days 10 and 11 of gestation, and there was no detectable μ- or m-calpain activity in these or younger embryos [31]. *Capn4*^-/- ^murine embryonic fibroblasts (MEFs) could be cultured from these embryos, although they also lacked calpain activity as assessed by casein zymography or by the formation of characteristic spectrin breakdown products, and they displayed migration defects consistent with a role for calpain in release of focal adhesions [9]. An independently derived *Capn4 *knockout, involving a more extensive deletion of the gene, resulted in an earlier embryonic lethality, apparently at a pre-implantation stage [32]. The different times of embryonic lethality suggested that the first reported *Capn4*^-/- ^mice [31] were targeted with a hypomorphic mutation, which retained some small level of calpain activity, allowing for their survival to mid-gestation, while the second reported *Capn4*^-/- ^mice [32] represented a true null mutation. Disruption of *Capn1*, encoding the μ-calpain catalytic subunit, was subsequently reported to result in fertile, viable mice with some mild defects in the μ-calpain rich platelets relating to their aggregation and clot retraction [33]. The fact that *Capn4 *null mice die during embryogenesis indicates that at least one of the ubiquitous calpains is essential for development to term. The viability of *Capn1*-deficient mice does not however distinguish between two possibilities: either that m-calpain is specifically required during embryogenesis, or that either form of calpain alone is sufficient and can compensate for the absence of the other. To resolve this question, we have now knocked out the *Capn2 *gene encoding the m-80 k subunit in mice. We report here that *Capn2 *null embryos died prior to the implantation stage, indicating that m-calpain is indispensable for early embryogenesis. This role cannot be fulfilled by μ-calpain, which is expressed in embryonic stem (ES) cells [31] and is assumed to be present at this stage of gestation. This demonstrates unequivocally that m-calpain and μ-calpain have distinct physiological roles during early embryogenesis.

## Results

### Isolation and characterization of *Capn2 *targeted ES cell clones

**Figure 2:**
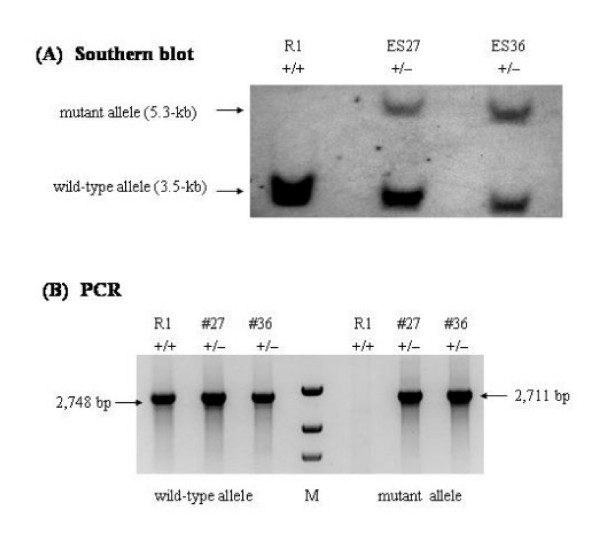
**Characterization of targeted ES cell lines**. (A) Targeted disruption of the *Capn2 *locus was detected initially by Southern blotting. Membranes were blotted with *Bam*HI-digested genomic DNA extracted from ES cells and hybridized with a DIG-labeled 823 bp *Bam*HI/*Hind*III fragment located immediately upstream of the short arm of the targeting vector (Figure 1). A 3.5-kb *Bam*HI fragment corresponding to the wild-type allele was present in all cells, whereas a 5.3-kb fragment from the mutant allele was detected in two targeted cell lines, designated ES27 and ES36. (B) PCR genotyping was carried out with two separate reactions designed to amplify either a 2,748 bp segment from the wild-type allele or a 2,711 bp segment from the mutant allele. Both reactions used a common sense primer located in intron 4, outside the short arm of the targeting vector, and distinct allele-specific antisense primers. The reaction to detect the wild-type allele used an antisense primer located in exon 7 while the amplification of the mutant sequence was done with an antisense primer in the PGK-Neo cassette. The results confirm the presence of the wild-type allele in all cells, whereas the mutant allele signal was observed only in the two targeted clones. (M) denotes the molecular weight marker.

Two independent *Capn2*^+/- ^ES cell lines, designated ES27 and ES36, were isolated from a screen of 305 drug-resistant clones. Correct targeting of the *Capn2 *locus was established both by Southern blot hybridization and PCR analysis. A probe located outside the short (upstream) arm of homology hybridized to a 3.5-kb *Bam*HI fragment of the wild-type allele and 5.3-kb *Bam*HI fragment of the mutant allele as predicted from genomic maps (Figure 2A). The same probe also detected the expected 7.2-kb wild-type and 6.4-kb mutant *Bgl*II fragments, 4.9-kb wild-type and 5.7-kb mutant *Nco*I fragments, as well as 7.2-kb wild-type and 4.9-kb mutant *Bgl*II/*Age*I fragments (not shown). A probe derived from the PGK-Neo cassette recognized only the 5.3-kb *Bam*HI fragment in *Capn2*^+/- ^ES cells, suggesting that the targeting vector had integrated solely at the *Capn2 *locus (not shown). A PCR screening method was also established that generated a wild-type product of 2,749 bp and a 2,711 bp product from the mutant allele. The 2,711 bp product was only evident in the two targeted cell lines (Figure 2B).

### Generation of chimeric mice and germline transmission of the mutant *Capn2 *allele

Eight chimeric male mice were produced in morula aggregation experiments using the *Capn2*^+/- ^ES27 cell line. Two of these males transmitted the *Capn2*^+/- ^ES27 genotype through the germline into the F_1 _generation. Heterozygous *Capn2*^+/- ^animals appeared normal, with no obvious defects in gross anatomy, reproduction, or life span.

**Figure 4:**
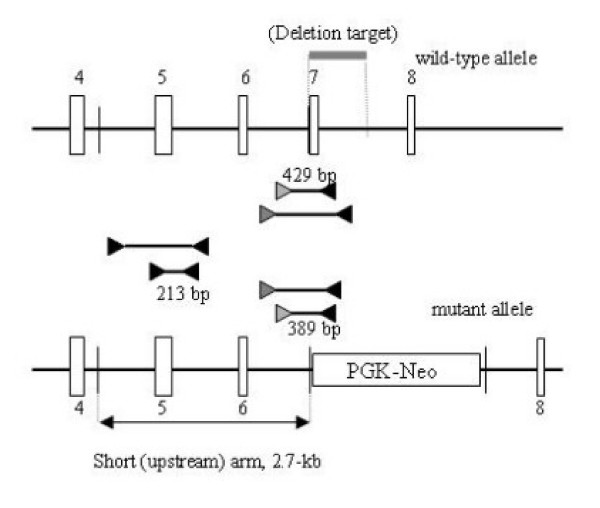
**Nested PCR strategy for genotyping of pre-implantation embryos**. Due to the scarcity of extractable genetic material, a nested PCR strategy was developed in order to genotype pre-implantation embryos. Separate reactions were used to amplify a 429 bp fragment from the wild-type allele and a 389 bp segment from the mutant allele, both spanning the 3' end of the short (upstream) arm of the targeting vector. In both reactions, a 213 bp sequence located within the short arm was co-amplified with the 'diagnostic' products as an internal control. The same sense primers were used to amplify 'diagnostic' sequences in both reactions, whereas the antisense primers were allele-specific. The primers, represented by triangles, are depicted in two (nested) sets for each of the three reactions. Exons are represented by open, vertical rectangles, the PGK-Neo cassette by an open, horizontal rectangle, while thin vertical lines denote the boundaries of the short arm and the 5' end of the long (downstream) arm. A grey, horizontal rectangle delineates the segment of the wild-type allele that is replaced by the PGK-Neo cassette in the mutant allele.

**Figure 5:**
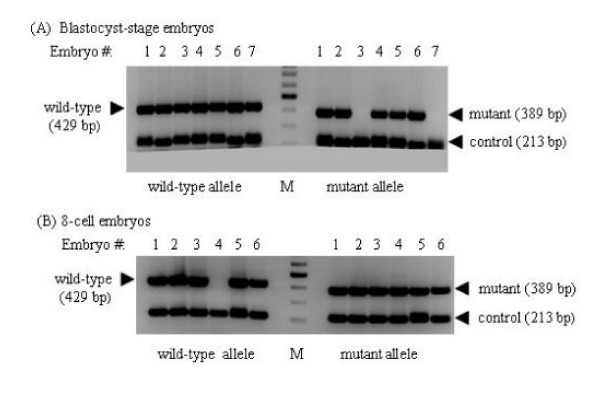
**Genotyping of pre-implantation embryos**. A nested PCR strategy was used to genotype embryos prior to implantation as described in Figure 4. *Capn2*^+/- ^mice were mated and the date of fertilization established by the appearance of vaginal plugs. Blastocyst (E3.5) or 8-cell embryos (E2.5) were flushed from the oviducts and then digested with proteinase K. In separate reactions segments found exclusively in either the wild-type or mutant alleles were co-amplified with an internal control sequence, located in the short (upstream) arm of the targeting vector, which is found in both alleles. The final products were 429 bp for the wild-type allele, 389 bp for the mutant allele, and 213 bp for the internal control. (A) A representative example of the genotyping of blastocyst stage embryos. Embryos #1, 2, 4, 5, and 6 were *Capn2*^+/- ^whereas embryos #3 and #7 were *Capn2*^+/+^, denoted by the absence of the 389 bp mutant signal. (B) An example of the genotyping of 8-cell embryos is shown. Embryos #1, 2, 3, 5, and 6 were *Capn2*^+/- ^while embryo #4 was *Capn2*^-/-^, marked by the absence of the 429 bp wild-type signal. (M) denotes the molecular weight marker.

Out of 199 weanlings from heterozygous intercrosses, no *Capn2*^-/- ^progeny were detected (Table 2). We did not observe high rates of perinatal death, and no *Capn2*^-/- ^stillborns were observed. This suggested that *Capn2*^-/- ^animals perished at some stage during embryonic development. In an attempt to determine if embryonic death occurred at a post-implantation stage, embryos were harvested for genotyping at different times between E10.5 and E18.5. No *Capn2*^-/- ^embryos were observed and no signs of embryo resorption were detected (Table 2). This indicated that the *Capn2*^-/- ^embryos might be dying prior to implantation. Embryos were then flushed from the oviducts of pregnant females at E2.5 or E3.5, and genotyped by means of a nested PCR strategy (Figure 4). Two of 90 successfully genotyped pre-implantation embryos were *Capn2*^-/-^, (Table 2; Figure 5). Both of these *Capn2*^-/- ^embryos were isolated at the 8-cell stage and did not display any obvious morphological defects. None of the 46 successfully genotyped blastocyst-staged embryos were *Capn2*^-/-^. The scarcity of *Capn2*-deficient embryos surviving to the 8-cell stage suggested that the loss of m-calpain activity must fatally compromise the viability of early embryos. Furthermore, it is possible that persistence of some maternally derived mRNA transcript or protein might have allowed a small number of *Capn2*^-/- ^embryos to survive to the morula-stage.

**Table 2:** Genotype distribution of offspring derived from *Capn2 *transgenic mice.

Cross	(N)	Age	Genotype			
			
			+/+	+/-	-/-	ND*
(+/-)♀ × (+/-)♂	199	3 weeks	23	176	0	0
(+/+)♀ × (+/-)♂	122	3 weeks	50	72	0	0
(+/-)♀ × (+/+)♂	103	3 weeks	28	75	0	0
						
(+/-)♀ × (+/-)♂	9	E18.5	0	7	0	2
(+/-)♀ × (+/-)♂	5	E17.5	0	5	0	0
(+/-)♀ × (+/-)♂	9	E14.5	2	7	0	0
(+/-)♀ × (+/-)♂	10	E11.5	3	7	0	0
(+/-)♀ × (+/-)♂	7	E10.5	2	5	0	0
						
(+/-)♀ × (+/-)♂	46	E3.5	7	39	0	0
(+/-)♀ × (+/-)♂	48	E2.5	8	34	2	4

* ND, not determined

The genotyping results for weanlings, post-implantation, and pre-implantation embryos are shown in Table 2. Curiously, the ratio of *Capn2*^+/+ ^to *Capn2*^+/-^animals from inbred heterozygous intercrosses was substantially less than the predicted 1:2 Mendelian ratio. In a group of 199 animals derived from heterozygote breeding (33 litters), 23 *Capn2*^+/+ ^(11.6%) and 176 (88.4%) *Capn2*^+/- ^animals were observed. The ratio of *Capn2*^+/+ ^to *Capn2*^+/- ^animals among males (14% to 82%) or females (13% to 90%) was essentially the same is it was for the combined population, and there were an average of six pups per litter, which is normal for this background strain. A larger than expected proportion *Capn2*^+/- ^animals was also evident in both post-implantation and pre-implantation embryos (Table 2). Interestingly, a similar over-representation of heterozygous animals was also reported in one of the *Capn4 *transgenic lines, though the genotype skewing was not as extreme [32]. Crosses between *Capn2*^+/+ ^and *Capn2*^+/- ^animals also resulted in a greater than expected proportion of *Capn2*^+/- ^animals (Table 2). An even higher degree of *Capn2*^+/- ^over-representation was seen when the mutant allele came the mother (73%) compared to when it came from the father (59%). In these crosses the ratios of *Capn2*^+/+ ^to *Capn2*^+/- ^animals among males or females compared well with the ratio in the combined populations; 77% of males and 69% of females were *Capn2*^+/- ^when the mutant allele came from the mother, and 62% of males and 55% of females were *Capn2*^+/- ^when the mutant allele came from the father.

### Attempted generation of *Capn2*^-/- ^ES cells

*Capn2*^+/- ^ES cells were subjected to clonal selection in the presence of 2 mg/mL G418 in attempts to generate homozygous mutant cells by gene conversion. This procedure has been extensively applied to targeted ES cells and was readily achieved in the case of *Capn4*^+/- ^ES cells [31]. In this case, however, no *Capn2*^-/- ^ES cells were isolated in screens of 126 drug-resistant clones. The inability to isolate *Capn2*^-/- ^ES cells, in concert with the absence of *Capn2*^-/- ^embryos beyond the 8-cell stage, suggested that m-calpain activity might be essential for cell viability or the establishment of viable ES cell clones.

## Discussion

Although calpain activity was first identified four decades ago, a clear understanding of the substrates and functions of the enzymes has remained elusive. In large part, this has been due to the lack of inhibitors capable of specifically down-regulating the calpains without affecting other proteases. In the past decade, the story has been further complicated by the discovery of a number of previously unknown isoforms which may be differently regulated and have different substrate specificity. Gene targeting in mice has provided a powerful approach to examine the physiologic roles of individual calpain isoforms. This was first used to disrupt the *Capn4 *gene, encoding the small regulatory subunit common to both μ- and m-calpain. Two independent laboratories observed embryonic lethality in *Capn4 *knockout mice, albeit at different stages of development [31,32]. These observations supported the hypothesis that the small subunit is required for both μ- and m-calpain, and furthermore suggested four possibilities regarding their requirement for embryonic development: 1) both isoforms were required; 2) μ-calpain was required; 3) m-calpain was required, or 4) μ- and m-calpain are redundant, and one or the other isoform was required. These options were narrowed down by the subsequent observation that *Capn1*^-/- ^mice, which lack the μ-calpain catalytic subunit, were healthy and fertile, although platelet aggregation and clot retraction defects were observed [33]. At that point, we were left with the last two possibilities that either m-calpain was specifically required during embryogenesis, or that either isoform alone was sufficient for sustaining embryonic viability.

We report here that *Capn2*^-/- ^mice, which lack the m-calpain catalytic subunit, die at the preimplantation stage of development. This observation allows us to now conclude that m-calpain is specifically required during embryogenesis. Since homozygous disruption of the *Capn4 *gene was also expected to abolish m-calpain activity, this result is in agreement with the phenotype presented by one of the two *Capn4 *targeted lines in which preimplantation lethality of null embryos was also observed [32]. The survival of *Capn4*^-/- ^embryos from the original targeted line reported by Arthur and colleagues to mid-gestation is more difficult to reconcile [31]. In retrospect, it seems likely that the latter line represents a hypomorphic state, rather than a true null. The *Capn4 *targeting strategy employed by Arthur and colleagues involved disrupting the C-terminus of the calpain small subunit by insertion of the PGK-Neo cassette into the middle of exon 9, which caused truncation of the protein [31]. This strategy was based upon previous structure/function studies showing that excision of the C-terminal 25 amino acid residues of the small subunit abolished calpain activity when co-expressed with the m-calpain large subunit in *E. coli *[34]. In contrast, the *Capn4 *targeting strategy employed by Zimmerman and colleagues involved a much more extensive deletion of exons 4 through 8 [32]. It now seems probable that the difference in the time of lethality of these two targeted *Capn4 *lines can be explained by different extents of disruption. The Zimmerman allele probably represents a true null genotype while the Arthur allele is likely a hypomorphic mutation. Alternate targeting strategies have been shown to yield different phenotypes in gene disruption studies. For example, three different targeting strategies were independently used to disrupt the mixed lineage leukemia (*Mll*) gene. In all three studies, homozygous null embryos perished during embryogenesis, but at different stages (E0.5, E10.5, E14.5) [35]. The variation in phenotype was attributed to the differences in degree of function of the truncated proteins produced from the mutant alleles. A similar effect might be at work in the two *Capn4 *transgenic lines. Efforts were made to detect transcripts or calpain activities derived from the Arthur *Capn4 *allele. This allele gave rise to multiple mRNA species, detectable by RT-PCR, reading through from the first half of exon 9 to at least two different cryptic splice sites in the PGK promoter sequence [31]. These transcripts could give rise to defective calpain small subunits with 10–30 inappropriate C-terminal acids, which might be sufficient to support a low level of calpain activity. However, expression of calpains with these modified small subunits did not give rise to any detectable calpain activity when expressed in *E. coli*, although their functionality in mammalian cells has yet to be determined (J.S. Elce, unpublished work) It has also been suggested that calpain large subunits alone might provide some activity in eukaryotic cells, although the Zimmerman et al. knockout appears to exclude that possibility, and no calpain activity was observed in our hands when calpain large subunits were expressed alone either in *E. coli *or in mammalian cells [36]. The different timing of lethality in the *Capn4 *knockouts might also be a consequence of the different genetic backgrounds of the two transgenic lines, which has been observed to influence the phenotype of transgenic mice on a number of occasions [37].

One of the enduring questions in calpain research has been whether the two ubiquitous isoforms, μ- and m-calpain, possess distinct *in vivo *roles. The two enzymes share 62% sequence identity and are very similar in their structure and biochemistry. Notably, they cleave essentially the same set of substrates *in vitro*, suggesting that they have the potential to carry out the same functions *in vivo*. On the other hand, since they require different amounts of Ca^2+ ^for *in vitro *activation, it is possible that the two isoforms are differentially regulated inside cells. It is now clear, from the gene targeting work done in mice, that μ- and m-calpain have some distinct physiological roles, at least during embryogenesis. As noted, whereas *Capn2 *null murine embryos die prior to implantation, homozygous disruption of the μ-calpain large subunit gene, *Capn1*, did not affect the viability of mice [33]. The principal phenotype observed as a result of *Capn1 *deficiency involved a disturbance in platelet function, possibly at the level of the tyrosine phosphorylation of certain proteins involved in platelet activation. Both μ- and m-calpain activities are present in most mammalian cells, although the published data, owing to weaknesses in the available methodology, do not provide reliable estimates of their relative amounts. Platelets and erythrocytes contain abundant μ-calpain activity, while m-calpain activity is barely detectable. Compensation for μ-calpain deficiency by m-calpain is therefore less likely in platelets and erythrocytes than in other cell types. As a result, it is not possible to determine whether the absence of marked phenotype in *Capn1*^-/- ^animals is due to a compensatory affect by the remaining m-calpain activity, or whether the functions of μ-calpain are simply not essential. In contrast, lethality in *Capn2*^-/- ^embryos demonstrates that m-calpain activity is essential for embryonic development beyond the 8-cell stage. It follows that at least some functions of μ-calpain and m-calpain are distinct.

The underlying cause of the preimplantation lethality in *Capn2*^-/- ^embryos has not yet been clarified. The two homozygous null embryos identified at the 8-cell stage did not present any obvious morphological defects. However, the fact that only two out of 90 successfully genotyped pre-implantation embryos proved to be *Capn2*^-/- ^is in itself revealing. Preimplantation lethality resulting from gene knockouts can typically be attributed to two general causes. In some cases, defects are incurred in the embryonic differentiation program which can often be observed morphologically [38]. Null embryos of this type often survive beyond the morula stage, and a Mendelian distribution of embryonic genotypes is usually noted. In other cases, however, the gene disruption is thought to compromise fatally the viability of cells in the early embryo [3,39-43]. In these instances, only a few null embryos are ever observed and homozygous mutant ES cells could not be isolated. The *Capn2 *knockout fits into the latter category. If it is true that m-calpain is essential for some aspect of cell viability, the survival of a few *Capn2*^-/- ^embryos to the 8-cell stage is most likely due to the persistence of some maternal m-calpain mRNA and/or protein through 2–3 cell divisions.

The mechanistic reasons for lethality in the absence of m-calpain are still unclear. There have been several reports of m-calpain involvement in cell proliferation in certain circumstances, including reports of its involvement in chromosome segregation during mitosis [44] as well as during meiosis [45]. Defects in migration, reported in *Capn4*^-/- ^cells [9], could contribute to failed embryonic development. The association between calpain and cell viability has been noted in *Capn4*^-/- ^MEFs and other cell lines, although the reported work frequently did not distinguish between μ- and m-calpain, and did not show that calpain was strictly essential [5,6]. In both CHO cells and *Capn4*^-/- ^MEFs, calpain was shown to influence cell proliferation, but only at very low cell densities [5]. Calpain has also been associated with progression through the G_1 _stage of the cell cycle [6]. Furthermore, some of the proteins known to be involved in cell cycle progression, such as p53, p107, cyclin D1, and p27^kip1 ^are reputed to be calpain substrates [3].

Sperm binding to the oocyte leads to increased cytoplasmic calcium which triggers the acrosome reaction [46]. Both μ- and m-calpain have recently been detected in rodent sperm [47] and oocytes [45]. m-calpain was implicated in the acrosome reaction [47] which correlated with a translocation of m-calpain to the cortical membrane in oocytes where it might participate in the release of cortical granule contents required to prevent polyspermy [45]. m-calpain also relocalized to the oocyte meiotic spindle after fertilization, were it could be involved in chromosome segregation [45]. Polyspermy or defective chromosome segregation would both have lethal effects on early embryonic development.

It should also be stressed that *Capn4*^-/- ^ES and MEF cells from the presumptive hypomorphic allele can be maintained in culture despite an apparent lack of calpain activity, as assessed by casein zymography or by the appearance of characteristic spectrin breakdown products. It is conceivable, as discussed earlier, that a trace amount of calpain activity, beneath levels detectable by these methods, is retained from this mutant *Capn4 *allele, and it is sufficient for maintaining the viability of the cells. Furthermore, calpain-independent mechanisms for protecting cell viability might exist in these cell lines that are absent in early embryonic cells.

The repeated failure to achieve gene conversion of *Capn2*^+/- ^to *Capn2*^-/- ^ES cells by selection of clones in high concentrations of G418 suggests that the homozygous mutant state somehow compromises cell viability or clonal selection of ES cells. Efforts are under way to express the m-80 k subunit from a *Capn2 *cDNA rescue transgene prior to gene conversion in order to preemptively rescue m-calpain activity before the remaining endogenous wild-type allele is lost. However, difficulty in achieving stable expression of the rescue transgene has thus far hampered these attempts.

One curiosity which arose during the genotyping of progeny from heterozygous interbreeding was the highly non-Mendelian ratio of *Capn2*^+/+ ^(11.6%) to *Capn2*^+/- ^(88.4%) of weanlings. At present, there is no obvious explanation for this result. Crosses between wild-type and heterozygous mice also produced progeny with a greater than expected proportion of heterozygous offspring. Interestingly, heterozygous crosses involving the *Capn4 *transgenic line generated by Zimmerman and colleagues yielded progeny with a similar, if less extreme, skewing in favor of the heterozygous genotype. Out of a total of 80 genotyped animals, 22.5% were wild-type and 77.5% were heterozygous, with no homozygous null progeny observed [32]. These observations suggest a developmental advantage associated with reduced calpain expression. Perhaps future studies will reveal a mechanistic basis for this.

## Conclusion

The work presented here has clarified two important questions regarding the physiological roles of the two ubiquitous calpain isoforms, μ- and m-calpain. Firstly, it was determined that m-calpain plays an indispensable role in murine embryogenesis, possibly related to pre-implantation development. Furthermore, this function cannot be carried out by μ-calpain despite the apparent *in vitro *similarities of μ- and m-calpain, demonstrating that the two isoforms clearly have some distinct roles *in vivo*. The functions of m-calpain during post-implantation embryogenesis and in adult mice remain to be elucidated and will have to be addressed using a conditional gene targeting strategy.

## Methods

### Cloning and sequencing of the mouse *Capn2 *locus

The mouse *Capn2 *gene encodes the 700 amino acids of the m-calpain large subunit (m-80 k) and consists of 21 exons extending over 50-kb on chromosome 1. A cDNA clone encoding a portion of the mouse m-80 k subunit was purchased (dbEST Id, 807416, Image:606689, Image Consortium, LLNL). It was found to contain 2.8-kb of sequence from position 247 of the coding sequence to the stop codon at position 2,101, including the 3'-UTR to the polyA signal. A fragment of this cDNA was subcloned and used to screen a 129Sv mouse genomic library in λ-Dash II. Overlapping genomic clones were obtained covering 15,354 bp, extending from a *Bam*HI site in intron 3 to a *Bam*HI site in intron 15. These clones were sequenced completely on both strands and the data were submitted to GenBank (accession no. AF497625). The submitted sequences agreed precisely with the public databases, and also filled in several small gaps corresponding to short repetitive sequences which could only be firmly established by repeated sequencing in non-standard conditions.

### Construction of the *Capn2 *targeting vector

**Figure 1:**
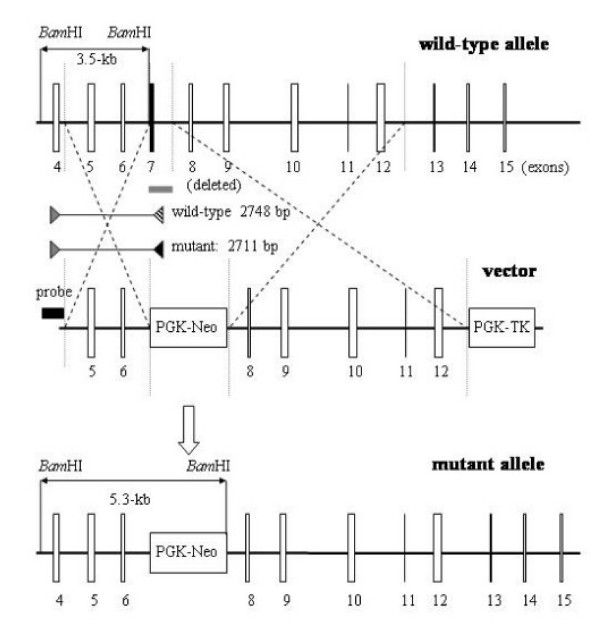
**Targeting strategy for disruption of the murine *Capn2 *gene**. The murine *Capn2 *gene, encoding the m-80 k subunit, was disrupted in ES cells by homologous recombination. The structures of the wild-type allele (top), the targeting vector (middle), and the mutant allele (bottom) are depicted. In the mutant allele, a PGK-Neo cassette replaces a 0.8-kb genomic fragment containing exon 7 (grey rectangle) which encodes the active site asparagine residue (Asn286) In the targeting vector, the PGK-Neo cassette is flanked by 2.7-kb of *Capn2 *homologous sequence in the upstream (short) arm and 7.9-kb of homology in the downstream (long) arm. A probe located immediately outside of the short arm detects a 3.5-kb *Bam*HI fragment from the wild-type allele and a 5.3-kb *Bam*HI fragment from the mutant allele. Exons are depicted as open vertical rectangles except for exon 7 which is represented by a solid vertical rectangle. The probe used in most Southern blot analyses is shown as a solid, horizontal rectangle, while triangles mark the positions of PCR primers also used for genotyping purposes.

A targeting construct was designed to replace a 785 bp *Bam*HI-*Hind*III fragment, containing exon 7 of the *Capn2 *gene, with the PGK-Neo cassette (Figure 1). Exon 7 encodes 24 amino acids in the active-site region, including Asn286, one of the catalytic triad residues. The short (upstream) arm of the targeting construct was provided by a 2.7-kb *Hin*dIII-*Bam*HI fragment, containing exons 5 and 6, which was inserted into the pPNT vector upstream of the PGK-Neo cassette [48]. During cloning, the *Bam*HI site at the 3' end of the short arm was abolished. The loss of this *Bam*HI site in the mutant allele, coupled with the introduction of a new *Bam*HI site at the 3' end of the PGK-Neo cassette, provided a basis for distinguishing the wild-type and mutant alleles by Southern blotting. The long (downstream) arm of homology was provided by a 7-kb *Hind*III-*Kpn*I fragment, extending from intron 7 to intron 12, inserted between the PGK-Neo and thymidine kinase *(tk) *cassettes.

### ES cell culture, transfection and selection of targeted clones

Mouse R1 ES cells [49] were maintained on gelatin-coated plates with feeder layers of mouse embryonic fibroblasts at 37°C under 5% CO_2 _in ES cell medium (DMEM [high glucose] supplemented with 15% fetal bovine serum, 0.1 mM non-essential amino acids, 2 mM glutamine, 1 mM sodium pyruvate, 0.1 mM 2-mercaptoethanol, 100 U/ml penicillin, 100 μg/ml streptomycin, 25 μg/ml amphotericin, and 1,000 U/ml of ESGro [Chemicon]). Fetal bovine serum from HyClone Laboratories Inc (Logan, Utah) was tested for its ability to support ES cell growth. Gelatin was from Sigma-Aldrich Canada (Oakville, Ontario). Unless otherwise specified, all other tissue culture reagents were from Gibco-BRL.

The targeting construct was linearized by *Not*I digestion and electroporated into R1 ES cells. Cells were plated without feeder layers on gelatin-coated plates and transformed clones were selected in the presence of 200 μg/ml G418 (Gibco- BRL) and 2 μM ganciclovir (Syntex, Inc.) for eight days. Drug-resistant clones were picked, expanded on gelatin-coated plates, and genotyped by Southern blotting and PCR analysis (see below).

### Generation of targeted mice

*Capn2*^+/- ^ES cells were aggregated overnight with 8-cell embryos recovered from CD1 matings, as previously described [31]. On the next day, blastocysts were transferred to pseudopregnant CD1 females. Chimeric animals were identifiable at birth by black eye pigmentation and subsequently by patches of agouti coat colour. Chimeric males were bred with CD1 females to identify those males capable of germline transmission. These were then bred with 129SvJ females to establish the mutation in an inbred genetic background. Mouse protocols were approved by the Queen's University Animal Care Committee according to the guidelines of the Canadian Council on Animal Care.

### Genotyping methods

Several Southern blot and PCR strategies were exploited in order to determine the genotype of the *Capn2 *locus. Southern blotting was carried out using the digoxigenin (DIG) non-radioactive system (Roche). In most cases, membranes were blotted with *Bam*HI-digested genomic DNA and hybridized with a DIG-labeled 823 bp exon 4-containing *Bam*HI-*Hind*III fragment located immediately upstream of the short arm of homology (Figure 1). A 681 bp *Pst*I-*Xba*I fragment from the PGK-Neo cassette was also used to probe Southern blots in order to verify a single integration event in targeted clones.

Genotyping was also carried out by PCR analysis of genomic DNA. The sequences of all oligonucleotide primers are listed in Table 1. A single-step PCR strategy was sufficient for genotyping ES cells or biopsies from post-implantation embryos and weanlings (Figure 1). A 2,748 bp segment of the wild-type allele and a 2,711 bp segment of the mutant allele were amplified in separate reactions using a common (intron 4) sense primer, located outside the short arm of homology, and distinct antisense primers which hybridized to either wild-type (exon 7) or mutant (PGK-Neo) sequence (Table I). The thermocycling parameters included a five minute initial denaturation step at 95°C, 30 cycles of one minute denaturation at 95°C, one minute annealing at 56°C, and one minute extension at 72°C, with a ten minute final extension step.

**Table 1:** Oligonucleotide primers used to genotype the *Capn2 *locus

Allele	Primer	Location	Oligonucleotide Sequence
Single-Step			
Both	Sense	Intron 4	5'-GGGCCCCCATTGCCTCTTAGC-3'
Wild-type	Antisense	Exon 7	5'- GGATTCCTGATGCGGATCAATTTCTGC-3'
Mutant	Antisense	PGK-Neo	5'-CCTCGAAGTCGAGGTCGATCC-3'
Nested PCR			
Wild-Type (Diagnostic)	Sense #1	Intron 6	5'-CAACATCATAAGCAACGGAGAACGC-3'
	Sense #2	Intron 6	5'-GCCTGTGACAGAAGTACCACCAG-3'
	Antisense #1	Intron 7	5'-CTCCTCGGCCCTCCCTGTAG-3'
	Antisense #2	Exon 7	5'-GGATTCCTGATGCGGATCAATTTCTGC-3'
			
Mutant (Diagnostic)	Sense #1	Intron 6	5'-CAACATCATAAGCAACGGAGAACGC-3'
	Sense #2	Intron 6	5'-GCCTGTGACAGAAGTACCACCAG-3'
	Antisense #1	PGK-Neo	5'-CCTACCCGGTAGAATTGACCTGC-3'
	Antisense #2	PGK-Neo	5'-GACCTGCAGGGGCCCTC-3'
			
Both (Internal Control)	Sense #1	Intron 4	5-GGGATGAAGGCTCCCTGTTGC-3'
	Sense #2	Exon 5	5'-GGGGGTGCCACCACGGAA -3'
	Antisense #1	Intron 5	5'-CCCCTAGAGGACTATTGCCTGG-3'
	Antisense #2	Intron 5	5'-GATGCACCCATGCCTTAAGGAGC-3'

Due to the limited amount of genetic material available in pre-implantation embryos, a nested PCR strategy was developed to yield reliable genotyping information (Figure 4). Whole embryos were first digested in 20 μL of proteinase K buffer (see below). The lysate was then divided in two, with half (10 μL) being used in the amplification of the wild-type allele and the remaining 10 μL in the amplification of the mutant allele.

The first reaction in the nested PCR amplification of the wild-type allele was carried out in a final reaction volume of 50 μL, using an intron 6 sense primer and an antisense primer located in intron 7. Two μL of the first reaction were used as template in the second PCR amplification using another intron 6 sense primer and an antisense primer in exon 7. The nested amplification of the mutant allele was carried out similarly. The first primer pair consisted of the intron 6 sense primer and an antisense primer in the PGK-Neo cassette. The nested primer pair was comprised of the second intron 6 sense primer and an antisense primer also located within the PGK-Neo sequence. It should be noted that amplification of both sequences involved the same sense primers in both steps of the nested PCR strategy. In addition, the two sets of reactions included a common internal control designed to amplify a genomic region within the short arm of the targeting vector that is preserved in both the wild-type and mutant alleles. The first primer pair of the control PCR was made up of an intron 4 sense primer and an intron 5 antisense primer. The second primer pair comprised an exon 5 sense primer and a nested intron 5 antisense primer. The final products were 213 bp for the control PCR, 429 bp for the wild-type PCR, and 389 bp for the mutant PCR. All reactions were carried out using identical PCR conditions entailing an initial five minute denaturation at 95°C, 35 cycles of one minute denaturation at 95°C, one minute annealing at 56°C, and one minute extension at 72°C, with a final extension step of ten minutes.

### Isolation of pre-implantation embryos

Time of fertilization was determined by observation of copulation plugs, and noon of that day was defined as E0.5. Pre-implantation embryos were obtained by dissecting the uteri out of pregnant females at E2.5 or E3.5 and flushing the oviducts with ES cell medium. Isolated embryos were then digested for five hours at 55°C in 20 μL of lysis buffer (50 mM Tris-HCl, 0.5% Triton X-100, 200 μL/mL proteinase K, pH 8.0), followed by ten minutes at 95°C to inactivate the proteinase K. Lysates were then used for PCR genotyping.

## List of abbreviations

EGF: Epidermal growth factor

ES: Embryonic stem

MEF: Mouse embryonic fibroblast

kDa: kilodalton

kb: kilobase

bp: base pair

## Authors' contributions

DEC generated the Capn2 targeting construct. PD and PAG carried out the ES electroporation and selection of targeted clones. KW performed aggregation chimeras to establish germline chimeric animals. TDV established and maintained the knockout mouse colony and performed genotyping analysis. PD, JSCA, JSE, DEC and PAG conceived the study and helped draft the manuscript. All authors read and approved the final manuscript.

**Figure 3:**
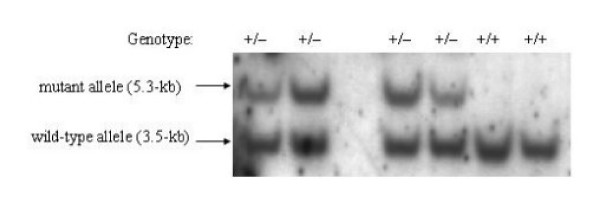
**Genotyping of weanlings from heterozygote intercrosses**. A representative example of the Southern blot genotyping of progeny from a *Capn2*^+/- ^intercross is shown. Genomic DNA was extracted from mouse tail biopsies taken from three-week old weanlings and genotyped by Southern blotting as described in Figure 1. A 3.5-kb *Bam*HI fragment from the wild-type allele was detected in all animals while a 5.3-kb *Bam*HI fragment from the mutant allele was observed in a subset of progeny. No *Capn2*^-/- ^offspring were detected among weanlings or post-implantation embryos, nor were embryo resorption sites observed. These results indicate that *Capn2*^-/- ^embryos perished at a pre-implantation stage.
